# FKBPL-based peptide, ALM201, targets angiogenesis and cancer stem cells in ovarian cancer

**DOI:** 10.1038/s41416-019-0649-5

**Published:** 2019-11-27

**Authors:** Stephanie Annett, Gillian Moore, Amy Short, Andrea Marshall, Cian McCrudden, Anita Yakkundi, Sudipto Das, W. Glenn McCluggage, Laura Nelson, Ian Harley, Nermeen Moustafa, Catherine J. Kennedy, Anna deFazio, Alison Brand, Raghwa Sharma, Donal Brennan, Sharon O’Toole, John O’Leary, Mark Bates, Ciarán O’Riain, Darran O’Connor, Fiona Furlong, Helen McCarthy, Adrien Kissenpfennig, Lana McClements, Tracy Robson

**Affiliations:** 1 School of Pharmacy and Biomolecular Sciences, Irish Centre for Vascular Biology, Royal College of Surgeons Ireland, Dublin, Ireland; 2 School of Pharmacy, Queen’s University Belfast, Belfast, UK; 3 Warwick Clinical Trials Unit, University of Warwick, Coventry, UK; 4 Department of Pathology, Belfast Health and Social Care Trust, Belfast, UK; 5 Northern Ireland Gynaecological Cancer Centre, Belfast Health and Social Care Trust, Belfast, UK; 6 Department of Gynaecological Oncology, Westmead Hospital, Sydney, NSW Australia; 7 Centre for Cancer Research, The Westmead Institute for Medical Research, Sydney, NSW Australia; 8 The University of Sydney, Sydney, NSW Australia; 9 NSW Health Pathology, ICPMR, Westmead, The University of Western Sydney, Westmead Hospital, Westmead, NSW Australia; 10 UCD School of Biomolecular and Biomedical Science, University College Dublin, Dublin, Ireland; 11 Department of Obstetrics and Gynaecology, School of Medicine, Trinity College Dublin, Dublin, Ireland; 12 Department of Histopathology, Trinity College Dublin, Dublin, Ireland; 13 Department of Histopathology, St. James’s Hospital, Dublin, Ireland; 14 The Wellcome-Wolfson Institute for Experimental Medicine, School of Medicine, Dentistry and Biomedical Sciences, Queen’s University Belfast, Belfast, UK; 15 The School of Life Sciences, Faculty of Science, University of Technology Sydney, Sydney, NSW Australia

**Keywords:** Ovarian cancer, Cancer stem cells, Tumour angiogenesis

## Abstract

**Background:**

ALM201 is a therapeutic peptide derived from FKBPL that has previously undergone preclinical and clinical development for oncology indications and has completed a Phase 1a clinical trial in ovarian cancer patients and other advanced solid tumours.

**Methods:**

In vitro, cancer stem cell (CSC) assays in a range of HGSOC cell lines and patient samples, and in vivo tumour initiation, growth delay and limiting dilution assays, were utilised. Mechanisms were determined by using immunohistochemistry, ELISA, qRT-PCR, RNAseq and western blotting. Endogenous FKBPL protein levels were evaluated using tissue microarrays (TMA).

**Results:**

ALM201 reduced CSCs in cell lines and primary samples by inducing differentiation. ALM201 treatment of highly vascularised Kuramochi xenografts resulted in tumour growth delay by disruption of angiogenesis and a ten-fold decrease in the CSC population. In contrast, ALM201 failed to elicit a strong antitumour response in non-vascularised OVCAR3 xenografts, due to high levels of IL-6 and vasculogenic mimicry. High endogenous tumour expression of FKBPL was associated with an increased progression-free interval, supporting the protective role of FKBPL in HGSOC.

**Conclusion:**

FKBPL-based therapy can (i) dually target angiogenesis and CSCs, (ii) target the CD44/STAT3 pathway in tumours and (iii) is effective in highly vascularised HGSOC tumours with low levels of IL-6.

## Background

Ovarian cancer affects one in 70 women in developed countries, and high-grade serous ovarian cancer (HGSOC) is the most common and aggressive subtype accounting for the majority of advanced cases.^1,2^ The 10-year survival is lower than 30% and this has not improved in 30 years, despite improved diagnostic and therapeutic interventions.^3^ The standard management consists of operative tumour debulking and administration of six cycles of paclitaxel and carboplatin chemotherapy.^4^ Approximately 80% of patients respond to first-line treatment; however, tumour recurrence and chemotherapy resistance eventually occurs in almost all patients within a median progression-free interval of 15 months post diagnosis.^4^

Angiogenesis has a pivotal role in the pathogenesis of ovarian cancer by promoting tumour growth and progression through ascites formation and metastatic spread.^5^ Targeting angiogenesis in ovarian cancer has been an active area of research, and bevacizumab, a monoclonal antibody against VEGF-A, has been approved by the European Medicines Agency (EMA) and recently the Food and Drug Administration (FDA) as a first-line therapy in combination with chemotherapy.^6,7^ This is based on the pivotal Phase III GOG-0218 trial in which those women who received bevacizumab in combination with chemotherapy had a median progression-free survival (PFS) of 18.2 months compared with 12.0 months in women who received chemotherapy alone (HR = 0.64; 95% CI 0.54–0.77, *p* < 0.0001).^8^ However, concerns regarding toxicity and resistance remain major hurdles for the clinical use of anti-angiogenic therapy. Across all tumour types, bevacizumab is discontinued in 8.4–22% of all patients due to adverse reactions.^9^ Furthermore, anti-angiogenic resistance, at least in part, is attributed to hypoxia-driven cancer stem cell (CSC) enrichment.^10^ It is now recognised that CSCs have major roles in the aetiopathogenesis, metastasis and chemoresistance of ovarian cancer and their targeting is an important therapeutic strategy.^11^ The successful elimination of CSCs could have unprecedented implications in the clinical management of patients.^12^

FK506-binding protein like (FKBPL) is a divergent member of the FK506-binding protein family first identified as having a role in the response of cells to radiation.^13,14^ At the intracellular level, and in a complex with Hsp90, FKBPL stabilises p21 and regulates oestrogen receptor (ER), androgen receptor and glucocorticoid receptor signalling.^15–18^ Furthermore, FKBPL demonstrated prognostic potential in a meta-analysis of five independent breast cancer TMA cohorts.^19^ FKBPL is also a secreted anti-angiogenic protein and the cell surface receptor, CD44, is a potential target for its activity.^20,21^ In support of a role for FKBPL in angiogenesis, FKBPL knockout mice are embryonically lethal and FKBPL heterozygous embryos display vascular irregularities, suggesting a critical role for FKBPL in developmental angiogenesis.^22^ In vitro and in vivo knockdown of FKBPL in breast cancer cell lines increases mammosphere formation accompanied by an increase in the pluripotency transcription factors (*Nanog*, *Sox2* and *Oct4*).^23,24^ Furthermore, FKBPL was identified by using an shRNA genetic screen library as a regulator of breast cancer tumour initiation,^25^ and high tumour *Fkbpl* and low *Nanog* are associated with improved survival outcomes in breast cancer patients (*n* = 94).^23^

The highly potent anti-angiogenic and anti-CSC activity of FKBPL is due to a unique sequence within the N-terminal region. A 24-residue peptide comprising amino acids 34–58 of FKBPL was developed and termed, AD-01. AD-01 has demonstrated potent anti-angiogenic and anti-CSC activity potentially through binding to CD44.^21,23^ Furthermore, FKBPL and its peptide derivatives inhibit breast cancer metastasis through Notch signalling.^26^ Analysis of the structure, activity and stability of AD-01 led to the selection of ALM201, a 23-residue peptide as the clinical drug candidate. ALM201 lacks cytotoxicity and displayed a very good safety profile in a Phase I, first-in-man, dose-escalation clinical trial in patients with ovarian cancer and other solid tumours (EudraCT number: 2014-001175-31).^27,28^ Furthermore, ALM201 was designated orphan drug status by the FDA in ovarian cancer. Given that anti-angiogenic agents are demonstrating efficacy in the HGSOC setting, a disease of unmet clinical need, we assessed whether ALM201 could elicit dual anti-angiogenic and anti-stemness activity in this disease. Indeed, this would differentiate this drug from other agents targeting angiogenesis only.

To begin addressing this, we investigated if ALM201 could target CSCs in a range HGSOC cell lines and patient samples. OVCAR3 cells were sensitive to ALM201 in vitro; however, xenograft studies indicated no antitumour or anti-CSC efficacy in vivo. On the other hand, Kuramochi xenografts demonstrated significantly reduced tumour growth and CSC frequency following ALM201 treatment. Further studies indicated differences in tumour vascularisation and cytokine levels between these two xenografts. OVCAR3 xenografts displayed extensive vasculogenic mimicry and limited CD31^+^ blood vessels, whilst Kuramochi xenografts had an extensive blood vessel network. In addition, OVCAR3 cells dramatically increased the expression of IL-6 in vivo and we demonstrated that IL-6 could inhibit the ability of ALM201 to target CSCs.

## Methods

### Tumoursphere assay

Briefly, 250 cells/cm^2^ were seeded in six-well dishes in non-adherent culture as described previously and treated once with ALM201 upon seeding.^29^ Tumourspheres > 50 µm were counted by using a Nikon Eclipse TE300 (Japan) microscope under ×4 magnification after 3–4 days for cell lines and 7 days for primary samples.

### Tumour initiation experiment

A total of 1 × 10^6^ OVCAR3 or 5 × 10^6^ Kuramochi cells were resuspended in PBS and diluted 1:1 in Matrigel (BD Bioscience, UK) and immediately implanted intradermally into female, 6-week-old, female SCID mice (Harlan Laboratories, UK). PBS (vehicle control) or ALM201 (0.3 mg/kg/day) were administered daily (d1–d5), from day 1, by subcutaneous injection (*n* = 5 mice/group) in the morning (between 9 am and 12 pm). Injections were conducted in a laminar flow to reduce infection risk. Tumour cells were implanted with the mice anaesthetised by using inhaled isoflurane (induction concentration 3–5% and maintenance concentration 1.25–3%) with an  anaesthetic machine and a face mask. Route of administration and dose were chosen based on previously conducted experiments by the lab.^21,23,26^ The mice were randomly allocated to experimental groups, with a weight range of 18– 22 g. Tumour volume was calculated as described previously.^23^ For all in vivo experiments, mice were housed in individually ventilated cages according to EU Directive 2010/63 at constant temperature and humidity with 12-h light/dark cycle and fed standard chow. The welfare of all the mice was monitored daily and health screening carried out regularly as per the policy of licensed establishment. Mice were euthanised by using exposure to carbon dioxide. No adverse events were noted for in vivo experiments. The experimental protocols were compliant with the UK Scientific Act of 1986 and ARRIVE guidelines (Supplementary Table 1) and Personal License Number 1598 under the Project License Number 2794.

### In vivo limiting dilution assay

SCID mice bearing Kuramochi xenografts from the above tumour initiation experiment were treated with PBS or ALM201 until tumours reached geometric mean diameter (GMD) of 12 mm^3^. Tumours were excised, disaggregated using a scalpel and added to a MACs C tube (Miltenyi Biotec, UK) containing collagenase type II (Invitrogen, UK), DNAase type 1 (Sigma-Aldrich, UK) in RPMI/1% penicillin/streptomycin (Invitrogen, UK). Tumours were minced by using a gentleMACS dissociator (Miltenyi Biotec, UK) and incubated at 37 °C in an orbital incubator for 45 min. The cell suspension was resuspended in red blood cell lysis buffer (Roche, UK) for 2–3 min. The cells were resuspended in ice-cold PBS and counted using a haemocytometer. Cells were implanted intradermally, as described above, into secondary SCID mice at 2.5 × 10^6^, 1 × 10^6^, 5 × 10^5^, 1 × 10^5^ and 1 × 10^4^ cells per mouse. Mice did not receive treatment and were observed for tumour initiation for 6 months. The tumour- initiating cell frequency was calculated by using ELDA software.^30^

### In vivo tumour growth delay

OVCAR3 and Kuramochi cells were implanted intradermally into SCID mice, as described previously. Established tumours (100 mm^3^) were then treated with PBS (vehicle control) or ALM201 (0.3 mg/kg/day) as described previously for 30 or 56 days in the OVCAR3 or Kuramochi xenografts, respectively (*n* = 5/group). Tumours were excised and used for downstream experiments.

### Tissue microarray

Individual patient data from four HGSOC tissue microarray (TMA) cohorts were obtained and summarised in Supplementary Table 2. TMAs were constructed at the various centres by using formalin-fixed, paraffin-embedded tissue from primary HGSOC with a 0.6-mm-diameter core (Cohort 1, 2, 3) or 1-mm- (Cohort 4) diameter core taken from tumour areas. Tissue staining was carried out at the Northern Ireland Molecular Pathology Laboratory of Queen’s University Belfast as described previously.^19^ TMAs were scored fully by one ‘trained’ scorer (SA/GM), with a second, independent scorer (SA/GM) evaluating a minimum of 20% of the cohort. Two cohorts were further independently scored by a clinical gynaecological pathologist (GMcC). Each scorer was blinded to all pathological information, and slides were scored according to staining intensity; only cores that consisted of >20% tumour were scored. A histoscore was calculated from the sum of (1 × % weakly positive tumour cells)  + (2 × % moderately positive tumour cells) + (3 × % strongly positive tumour cells) with a maximum histoscore of 300 as described in ref. ^19^ and sent to the independent statistics team at the University of Warwick for analysis.

## Results

### The FKBPL-derived therapeutic peptide, ALM201, targets CSCs in HGSOC cell lines and patient samples

**Fig. 1:**
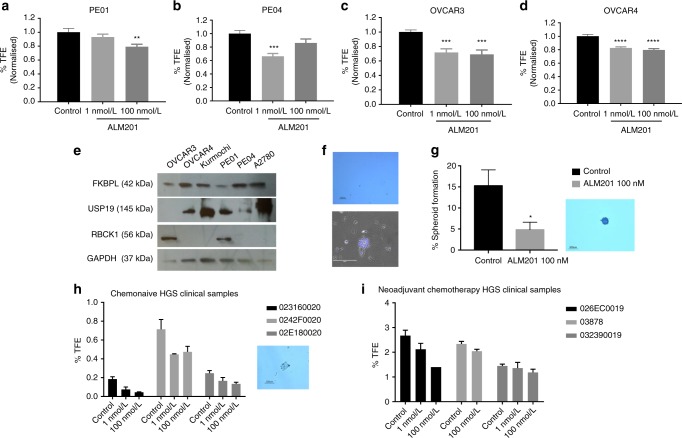
FKBPL and its clinical peptide derivative, ALM201, reduce tumoursphere formation in ovarian cancer cell lines and high-grade serous patient samples. The effect of ALM201 treatment on the primary TFE in the **a** PE01, **b** PE04, **c** OVCAR3 and **d** OVCAR4 after treatment with 1 and 100 nmol/L ALM201 treatment. **e** Protein expression of FKBPL, USP19 and RBCK1 was analysed in ovarian cell lines (OVCAR3, OVCAR4, Kuramochi, PE01, PE04 and A2780) by western blot (*n* = 3). **f** The Kuramochi cell line does not form tumourspheres (top picture) and Kuramochi PGCCs were isolated by incubation with cobalt chloride (450 μM) for 72 h (bottom picture). **g** The Kuramochi PGCCs were trypsinised and seeded into Matrigel containing tumoursphere media and treated with PBS or ALM201 (100 nmol/L). A representative image of spheroid formed from a PGCC (inset). Cells were incubated for 3 weeks, with fresh ALM201 added weekly and the number of spheroids >50 μM counted manually. **h** Tumoursphere formation of cancer cells derived from primary chemo-naive high-grade serous ovarian tumours (*n* = 3) and **i** primary high-grade serous ovarian tumours that received neoadjuvant chemotherapy (*n* = 3). Data points are mean ± SEM. *n* ≥ 3. *P < 0.05; **P < 0.01; ***P < 0.001 (one-way ANOVA or two-tailed Student *t*-test). TFE, tumoursphere-forming efficiency.

The tumoursphere assay was used to assess the ability of ALM201 to reduce ovarian CSCs in vitro and ex vivo. A significant reduction in tumoursphere-forming efficiency (TFE) of 20–30% was obtained across all cell lines, PE01, PE04, OVCAR3 and OVCAR4 cell lines (Fig. 1a–d), similar to what we had observed in breast cancer cell lines with the preclinical peptide, AD-01.^23^ FKBPL levels were assessed in all cell lines, with highest expression observed in OVCAR4 cells and lowest expression in PE01 cells (Fig. 1e). There was no difference between endogenous FKBPL levels and the response of the cell lines to ALM201 in the tumoursphere assay (Fig. 1e, Supplementary Fig 1). RBCK1 is an FKBPL-interacting protein, which regulates FKBPL stability at the post-translational level via ubiquitination.^31^ RBCK1 was also measured in the ovarian cancer cell lines. Again, there was no correlation between RBCK1, USP19 and FKBPL in the ovarian cancer cell lines (Fig. 1e, Supplementary Fig 1). The Kuramochi cell line, reported to closely resemble HGSOC,^32^ did not form tumourspheres (Fig. 1f). However, polypoid giant cancer cells (PGCCs) were routinely observed in the Kuramochi monolayer (Fig. 1f). PGCCs are induced by hypoxia or chemotherapy and they generate daughter cells with CSC-like properties through an evolutionary conserved, asymmetric budding mechanism. Zhang et al. reported that spheroids derived from PGCCs are positive for CSC markers and a single PGCC spheroid from the ovarian HEY cell line was able to form tumours in vivo.^33^ Encouragingly, ALM201 (100 nmol/L) significantly reduced the number of spheroids formed, suggesting a reduction in the tumour-initiating population in the Kuramochi cell line (Fig. 1g). The anti-CSC activity was further evaluated by using clinically relevant fresh primary HGSOC tissue directly from patients (Supplementary Table 3). Treatment with ALM201 (1 and 100 nmol/L) was able to reduce the number of tumourspheres representative of CSCs in three chemo-naive samples by ~40% (Fig. 1h, Supplementary Fig. 2a). Neoadjuvant chemotherapy is reserved for patients with aggressive tumours for whom optimal tumour debulking is not possible.^34^ Patients who received neoadjuvant chemotherapy demonstrated an approximately ten-fold increase in the TFE compared with chemo-naive patients (Fig. 1h, i). However, ALM201 also reduced CSCs in the neoadjuvant patients, albeit with a lower average reduction of ~20% TFE (Fig. 1i, Supplementary Fig. 2b). Upon grouping the patient samples, treatment with ALM201 significantly inhibited tumoursphere formation in chemo-naive patients, but not in the neoadjuvant patients (Supplementary Fig. 2). On the whole, ALM201 appears to effectively reduce tumoursphere formation in chemo-naive HGSOC, indicating that it may be more effective as a first-line agent. ALM201 demonstrates a mixed anti-CSC response in other subtypes of ovarian cancer, with clear activity in the A2780 cell line (endometrioid like cell line), an endometrioid patient, adenocarcinoma patient, clear-cell patient and a serous borderline patient (Supplementary Fig. 3).

**Fig. 2:**
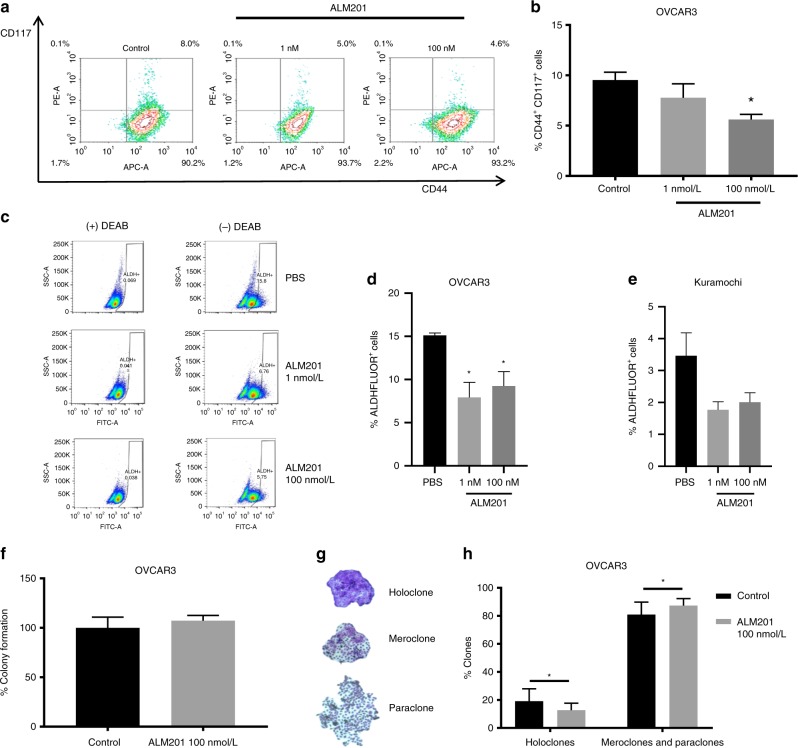
ALM201 reduces the CD44^±^/CD117^±^ and ALDH^±^ cell subpopulation by differentiating the CSCs to more ‘mature’ cancer cells. **a** Representative flow cytometry images demonstrating a reduction in the CD44^+^/CD117^+^ subpopulation following 72-h ALM201 treatment of OVCAR3 monolayers. **b** Percentage of CD44^+^/CD177^+^ OVCAR3 cells after treatment with ALM201 compared with PBS-treated controls. **c** Representative flow cytometry images demonstrating a shift in the ALDH^+^ cell population in OVCAR3 cells after treatment for 72 h with ALM201 (1 and 100 nmol/L). **d** Percentage reduction in the ALDH^+^ cell population was quantified in OVCAR3 and **e** Kuramochi cell lines following treatment for 72 h with ALM201 (1 and 100 nmol/L). **f** ALM201 treatment does not affect the total number of colonies formed. **g** Representative images of OVCAR3 colonies: holoclones, meroclone and paraclones; different colonies were manually counted and expressed per 100 cells seeded. **h** A reduction in the number of holoclones formed and a concomitant increase in the number of more differentiated, meroclone and paraclone colonies, following ALM201 treatment was observed in OVCAR3 cells. Data points are mean ± SEM. *n* ≥ 3. *P < 0.05; **P < 0.01 (one-way ANOVA or two-tailed Student *t*-test). SSC, side scatter.

To validate the tumoursphere assays, we used flow cytometry to quantitate the ALM201-mediated reduction in ovarian CSCs using well-characterised ovarian CSC surface markers, CD44^+^/CD117^+^.^35^ There was a significant decrease in the CD44^+^/CD117^+^ subpopulation in OVCAR3 cells following ALM201 (100 nmol/L) treatment (Fig. 2a, b). The Kuramochi cell line had no detectable CD44^+^/CD117^+^ subpopulation. The ALDEFLUOR assay was also used to analyse the effect of ALM201 on the ALDH^+^ subpopulation, which is also representative of ovarian CSCs.^36^ There was a significant decrease in OVCAR3 ALDH^+^ cells following ALM201 (1 and 100 nmol/L) treatment (*p* < 0.05; *n* = 5) and a decrease of Kuramochi ALDH^+^ cells following ALM201 treatment, but this was not significant (*n* = 4) (Fig. 2c–e). There was an average of 15.1% ALDH^+^ cells in the OVCAR3 cell line compared with 3.46% in the Kuramochi cell line (Fig. 2d, e). Together, this indicates that the stem cell-like population is small in the Kuramochi in vitro population, given the lack of ability to form tumourspheres, no detectable CD44/^+^CD117^+^ subpopulation and a small ALDH^+^ subpopulation.

In order to investigate the fate of CSCs following treatment with ALM201, we assessed colony morphology by using a clonogenic assay. By using this assay, we have previously reported that the preclinical peptide, AD-01, was not cytotoxic but rather differentiated breast CSCs into a more committed cell phenotype.^23^ Similar to what was observed with AD-01, ALM201 was not cytotoxic (Fig. 2f) and it significantly reduced holoclone formation and increased meroclone and paraclone formation (Fig. 2g, h). These results further support the hypothesis that ALM201 differentiates CSCs into more ‘mature’ cancer cells.

### ALM201 does not target CSCs or angiogenesis in OVCAR3 xenografts

**Fig. 3:**
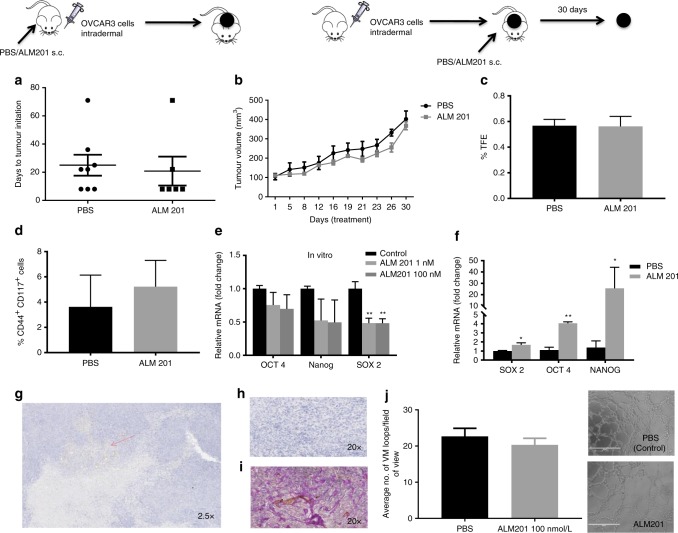
ALM201 does not target CSCs or inhibit angiogenesis in OVCAR3 xenografts. **a** Tumour initiation in vivo assay following implantation of OVCAR3 cells and treatment with PBS or ALM201 (0.3 mg/kg/day) subcutaneously from day 1 (inset experimental design; *n* *=* 5). **b** OVCAR3 cells were implanted into mice, tumours established until 100 mm^3^ and then treated with PBS or ALM201 (0.3 mg/kg/day) for 30 days (inset experimental design; *n* = 5). **c** OVCAR3 tumour xenografts were excised and dissociated, and the CSC subpopulation analysed by tumoursphere assay (*n* = 3) or **d** flow cytometry by quantifying CD44^+^CD117^+^ cell population (*n* = 3). **e** OVCAR3 monolayers were treated with ALM201 (1 and 100 nmol/L) for 24 h, and expression levels of the pluripotency transcription factors (*SOX2*, *OCT4* and *NANOG*) analysed by q-PCR. **f** Expression of pluripotency transcription factors in OVCAR3 xenografts following treatment with PBS or ALM201 (0.3 mg/kg/day) for 30 days. **g** OVCAR3 xenografts were sectioned, and immunohistochemistry staining for CD31^+^ blood vessels conducted. A small number of blood vessels (red arrow) were observed at ×2.5 magnification and no CD31^+^ vessels (**h**) in the majority of the xenografts at ×20 magnification. **i** CD31^+^/PAS dual immunohistochemistry staining of OVCAR3 indicated extensive vasculogenic mimicry networks in OVCAR3 xenografts. **j** Treatment with ALM201 (100 nmol/L) does not inhibit OVCAR3 tubule formation (representative image in inset; *n* = 3). Each dot represents a single mouse. Data points are mean ± SEM. *n* ≥ 3. *P < 0.05; **P < 0.01 (one-way ANOVA or Student *t*-test).

To validate the antitumour activity of ALM201 in vivo, a tumour initiation experiment was performed by using the OVCAR3 xenograft model. Mice were treated with ALM201 (0.3 mg/kg/day) from day 1 of implantation. Surprisingly, ALM201 did not delay tumour initiation of the OVCAR3 xenografts (Fig. 3a). We then used a tumour growth delay model to investigate the ability of ALM201 to inhibit angiogenesis. Established (100 mm^3^) OVCAR3 xenografts were treated with ALM201 (0.3 mg/kg/day; d1–d5). No significant delay in tumour growth was observed either, suggesting that ALM201 does not inhibit angiogenesis in this model (Fig. 3b). Following 30 days of treatment, tumours were excised and dissociated. The dissociated OVCAR3 xenograft cells were assessed in an ex vivo tumoursphere assay, and no decrease in TFE was observed in the ALM201 treatment group (Fig. 3c). In addition, flow cytometry was conducted and ALM201-treated xenografts demonstrated no significant decrease in the CD44^+^CD117^+^ CSC-like subpopulation (Fig. 3d). Overall, these results indicate that ALM201 does not target CSCs or angiogenesis in OVCAR3 xenografts. We had previously shown that the preclinical peptide, AD-01, significantly decreased the mRNA expression of pluripotency markers *OCT4*, *NANOG* and *SOX2* in breast cancer xenografts, consistent with the differentiation of the CSCs.^23^ Likewise, *Sox2* mRNA levels were significantly reduced in OVCAR3 monolayer cells after 24-h in vitro treatment with ALM201 (1 and 100 nmol/l) (Fig. 3e). However, *Oct4*, *Nanog* and *Sox2* mRNA levels were significantly increased in the ALM201-treated xenografts, consistent with the lack of anti-CSC activity in the tumour xenograft setting (Fig. 3f). The OVCAR3 xenografts were sectioned and stained for CD31^+^ blood vessels. Not surprisingly, given the lack of any significant antitumour efficacy in this xenograft model, there were very few CD31^+^ blood vessels in the OVCAR3 xenografts (Fig. 3g, h), and so we considered that other methods of vascularisation were driving growth. The xenografts were dual stained with CD31^+^/PAS^+^, markers for vasculogenic mimicry (VM). An extensive network of PAS^+^ vessels was observed in the xenografts, suggesting a non-angiogenic tumour phenotype (Fig. 3i). An in vitro model of VM was evaluated, by inducing tubule formation in OVCAR3 cells. There was no difference in tubule formation after ALM201 (100 nmol/L) treatment in OVCAR3 cells (Fig. 3j). Together, these data indicate that OVCAR3 xenografts induce VM channels for tumour growth and this cannot be inhibited by ALM201 (Fig. 3). The Kuramochi cells do not form tubules in vitro (Supplementary Fig. 4).

### ALM201 targets CSCs and angiogenesis in Kuramochi xenografts

**Fig. 4:**
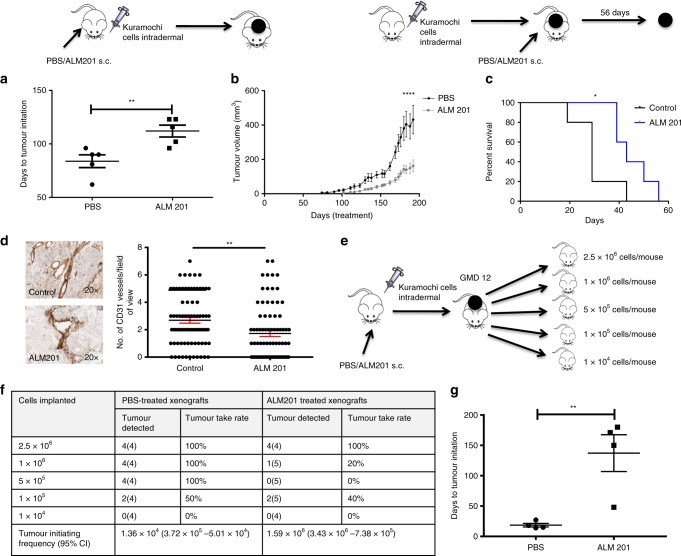
ALM201 targets CSCs and angiogenesis in the Kuramochi xenografts. **a** In vivo tumour initiation assay following implantation of Kuramochi cells into SCID mice and subcutaneous treatment with PBS or ALM201 (0.3 mg/kg/day) from day 1; days to tumour initiation were calculated (inset experimental design; *n* = 5) **b** and tumour growth monitored. **c** Kuramochi cells were implanted into mice, tumours established until 100 mm^3^ and treatment with PBS or ALM201 (0.3 mg/kg/day) administered for 56 days (inset experimental design; *n* = 5). ALM201 treatment increased survival, as determined by time to tumour quadrupling. **d** Kuramochi xenografts were sectioned, and immunohistochemistry staining for CD31^+^ blood vessels conducted, and ALM201 significantly decreased the number of blood vessels. **e** Tumour cells dissociated from ALM201 or PBS-treated Kuramochi xenografts were re-implanted in a limiting dilution assay into second-generation mice. The second-generation mice did not receive further treatment and were observed for tumour initiation. **f** The number of mice that developed tumours after 6 months of monitoring is tabulated. Tumour-initiating frequency (TIF) was calculated by using ELDA software (*p* = 8.77 × 10^−5^; *n* > 4/group). **g** In total, 2.5 × 10^6^ cells were re-implanted into second-generation mice, and tumours from first-generation ALM201-treated mice demonstrated a 118-day delay in tumour initiation. Each dot represents one mouse. Data points are mean ± SEM. *n* ≥ 3. *P < 0.05; **P < 0.01 (two-tailed, unpaired *t*-test or one-way ANOVA).

A tumour initiation experiment was performed by using Kuramochi cells, and a significant 28-day delay in tumour initiation and subsequent delay in tumour growth was observed in the ALM201 (0.3 mg/kg/day) treatment group (*n* > 5) (Fig. 4a, b). This was also reflected in the Kaplan–Meier survival curves (Fig. 4c). Kuramochi xenografts from mice treated with PBS or ALM201 (0.3 mg/kg/day) were then stained for CD31^+^ blood vessels. Unlike the OVCAR3 xenografts, Kuramochi xenografts demonstrated a robust vascular network, and there was a significant decrease in CD31^+^ vessels after treatment with ALM201, indicating a reduction in angiogenesis (Fig. 4d). Interestingly, the Kuramochi tumour cells also stained positive for CD31 (Fig. 4d). The in vivo limiting dilution assay is the gold standard for assessing agents that target the tumour-initiating potential of CSCs. Kuramochi xenografts were treated with ALM201 (0.3 mg/kg/day) until a GMD = 12. Tumours were then disaggregated and implanted into second-generation mice at defined cell numbers (2.5 × 10^6^, 1 × 10^6^, 5 × 10^5^, 1 × 10^5^ and 1 × 10^4^ cells/mouse, Fig. 4e). The second-generation mice did not receive ALM201 treatment, and extreme limiting dilution analysis (ELDA) software was used to estimate the frequency of tumour-initiating cells in the xenografts.^30^ There was a greater than ten-fold decrease in the tumour-initiating frequency (TIF) in untreated second-generation xenografts derived from primary ALM201 treatment mice compared with the PBS controls (TIF; PBS 1.36 × 10^5^ vs. ALM201 1.59 × 10^6^; *p* = 8.77 × 10^5^, *n* > 4) (Fig. 4f; Supplementary Fig. 5). In addition, there was a dramatic 131.5-day delay in tumour initiation between mice implanted with 2.5 × 10^6^ cells previously treated with ALM201 and mice implanted with 2.5 × 10^6^ cells from PBS-treated xenografts (Fig. 4g). These results strongly indicate that ALM201 is highly effective at targeting both the CSC subpopulation and angiogenesis in the highly vascularised Kuramochi xenografts.

### The Kuramochi cell line displays a pro-angiogenic genotype compared with OVCAR3 cell line

**Fig. 5:**
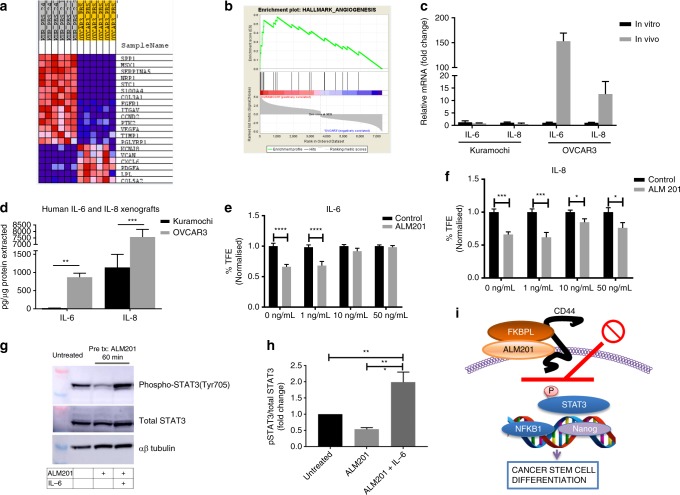
OVCAR3 xenografts upregulate inflammatory cytokines and ALM201 anti-CSC activity is abrogated by IL-6. **a** Heat map and **b** enrichment plot of angiogenesis-related genes upregulated (red) in Kuramochi cell line compared with the OVCAR3 cell line by RNAseq analysis. **c** IL-6 and IL-8 mRNA is upregulated in vivo compared with in vitro in the OVCAR3 cells but not in the Kuramochi cells (*n* > 3). **d** Human IL-6 and IL-8 protein is significantly higher in the OVCAR3 xenografts compared with the Kuramochi xenografts; mouse IL-6 and IL-8 (Kc) was not detected. **e** Addition of recombinant IL-6 to OVCAR3 tumoursphere assay abrogated the ability of ALM201 to decrease tumourspheres (*n* = 3). **f** ALM201 decreases OVCAR3 tumourspheres in the presence of IL-8. **g** Representative western blot demonstrating that ALM201 decreases phosphorylation of STAT3 and this effect is abrogated by addition of IL-6. **h** Densitometric analysis of western blots by using ImageJ, *n* ≥ 3. **i** Diagram summarising the effect of ALM201 on OVCAR3 cancer cells. Data points are mean ± SEM. *n* ≥ 3. *P < 0.05; **P < 0.01 (two-way ANOVA).

RNA sequencing was performed to investigate gene expression differences between the untreated Kuramochi and OVCAR3 cell lines. The Kuramochi cell line demonstrated a positive correlation to angiogenesis gene regulation, including an upregulation of VEGF-A, compared with the OVCAR3 cell line (Fig. 5a, b). Other pathways that had differential expression between Kuramochi and OVCAR3 cells included p38MAPK, TGFβ, mTOR and NOD-like receptor signalling (Supplementary Fig. 6). These data support the well-vascularised phenotype observed when Kuramochi cells were grown as xenografts and the distinct lack of angiogenesis when OVCAR3 cells were grown as xenografts.

### The OVCAR3 cell line upregulated inflammatory cytokines in vivo, which inhibited anti-stem cell activity of ALM201

A previous study, by using unsupervised hierarchical clustering of HGSOC patients treated with bevacizumab, a VEGF inhibitor, identified three major subgroups: two with angiogenic gene upregulation and one subgroup with immune gene upregulation.^37^ The OVCAR3 and Kuramochi in vitro monolayers had similar mRNA expression of the pro-inflammatory cytokines IL-6 and IL-8 (Fig. 5c). However, there was a dramatic 150-fold increase in IL-6 and 12.5-fold increase in IL-8 mRNA levels when OVCAR3 cells were grown as xenografts (Fig. 5c). Notably, there was no change in IL-6 and IL-8 mRNA levels between the Kuramochi cell line cultured as a monolayer or as xenografts (Fig. 5c). The levels of both mouse and human IL-6 and IL-8 (Kc) in the OVCAR3 and Kuramochi xenografts were measured by ELISA. Mouse IL-6 or IL-8 was undetectable (data not shown), indicating that the source of the cytokines was tumour derived rather than being from stromal tissue. The Kuramochi xenografts had low levels of IL-6 (17 pg/µg), while the OVCAR3 xenografts had 51-fold more IL-6 (871 pg/µg, Fig. 5d). The OVCAR3 xenografts also had approximately six-fold more IL-8 (Kc) protein than the Kuramochi xenografts (Fig. 5d). This suggests that the OVCAR3 cell line is more representative of an immune subgroup, whilst the Kuramochi cell line is representative of an angiogenic subgroup of HGSOC.

We had previously shown that ALM201 targets the CSC subpopulation in the OVCAR3 cells in in vitro assays (Figs. 1, 2) but had no anti-CSC activity in in vivo OVCAR3 xenografts (Fig. 3). We decided to evaluate whether the increased IL-6 and IL-8 in OVCAR3 xenografts could explain the lack of response to ALM201. Recombinant IL-6 and IL-8 was added to in vitro OVCAR3 tumoursphere assays in the presence of ALM201. IL-6 significantly abrogated the ability of ALM201 to decrease tumoursphere formation at concentrations >10 ng/ml (Fig. 5e). However, ALM201 was still able to reduce tumoursphere formation in the presence of IL-8 (Fig. 5f), suggesting that IL-6, a known antagonist of other anti-CSC and anti-angiogenic drugs, might be responsible for the lack of ALM201 anti-CSC efficacy in OVCAR3 xenografts.^38,39^

### ALM201 decreases phosphorylation of STAT3 in OVCAR3 cells

The principal signalling mechanism for IL-6 is via the JAK/STAT pathway. Here we addressed whether IL-6 could abrogate ALM201 activity via inhibiting this pathway. We first investigated whether ALM201 could inhibit phosphorylation of STAT3, a pathway also associated with CD44 signalling.^40^ Indeed, ALM201 decreased p-STAT3(Tyr705) in OVCAR3 cells, whilst recombinant IL-6 abrogated its activity post treatment with ALM201 (Fig. 5g, h). We have previously reported that FKBPL and its peptide derivatives might exert their activity though the cell surface receptor, CD44. STAT3 forms a complex with CD44 in the cytoplasm and acts as a linker molecule to NFκB signalling to promote the CSC phenotype.^40,41^ Therefore, to further support a role for FKBPL in this pathway, we demonstrate that transient knockdown of *Fkbpl* in OVCAR3 cells resulted in the transient upregulation of *NFĸB1* and the pluripotency factor *NANOG* (Fig. 5i, Supplementary Fig. 7).

### High FKBPL expression in ovarian cancer is associated with an increase in progression-free survival

**Fig. 6:**
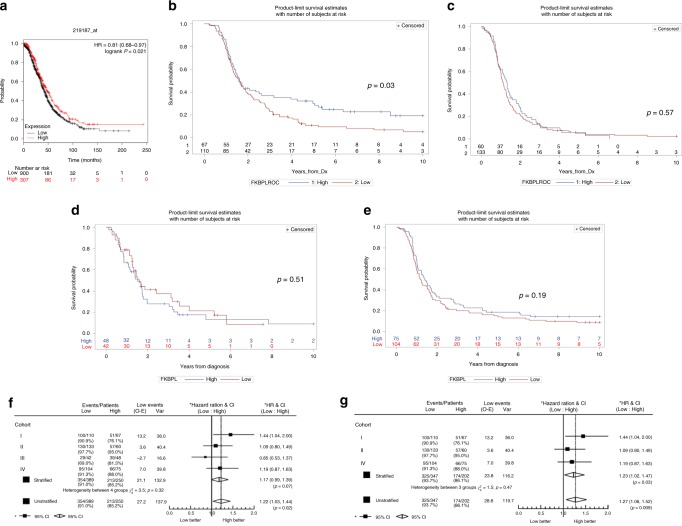
High FKBPL expression increased progression-free survival. **a** FKBPL expression was analysed by using microarray data from publicly available data sets (http://www.kmplot.com/ovar). Kaplan–Meier survival curves of ovarian cancer patients were generated, showing that those with low mRNA FKBPL expression indicated a significantly reduced overall survival (*p* < 0.05). FKBPL expression Kaplan–Meier estimates of HGSOC PFS from diagnosis in cohort I (*n* = 177; **b**), cohort II (*n* = 193; **c**), cohort III (*n* = 99; **d**) and cohort IV (*n* = 180; **e**). Kaplan–Meier estimates were determined with average FKBPL score for PFS, where FKBPL protein expression has been separated by histoscore of 190: high >190 (blue) and low < 190 (red). **f** Hazard ratio plot of HGSOC PFS from diagnosis against FKBPL levels by cohorts I, II, III and IV (*n* = 639). **g** Hazard ratio plot of HGSOC PFS from diagnosis against FKBPL levels by cohort from cohorts I, II and IV (*n* = 549).

A meta-analysis of five breast cancer TMA cohorts has previously indicated that FKBPL is an independent marker of good prognosis in breast cancer,^19^ not surprising given its anti-angiogenic and anti-CSC activity in this setting.^19,21,23^ Here we have demonstrated that the FKBPL peptide mimetic, ALM201, has antitumour activity in HGSOC and therefore postulated that FKBPL might also be a prognostic marker in this setting. The association of FKBPL expression with overall survival was assessed within publicly available data sets. Analysis of 1582 ovarian cancer patients of all subtypes and treatments demonstrated that low FKBPL expression was significantly associated with reduced overall survival (*p* = 0.021) (Fig. 6a). This preliminary data suggested a significant correlation between reduced mRNA FKBPL expression and reduced overall survival, correlating with what was observed in breast cancer.^16^ We then used four TMA cohorts from HGSOC patients to determine if FKBPL levels were associated with prognosis in this tumour type. The patient clinico-pathological variables for all four cohorts are shown in Supplementary Table 2. Receiver-operative characteristics (ROC) analysis was carried out on cohort I and II, and a histoscore of 190 was determined to be the optimum cut-off (Supplementary Fig. 8). A histoscore of 190 was also previously used as the cut-off in five breast cancer TMAs^19^ and was therefore considered a suitable cut-off for this analysis. In cohort I, there was a significant association between high FKBPL and progression-free survival (PFS; *p* = 0.03, HR = 1.44 and 95% CI = 1.04–2.00; Fig. 6b). However, whilst there was a trend for high FKBPL levels demonstrating improved PFS, this was not significant in cohorts II (Fig. 6c), III (Fig. 6d) and IV (Fig. 6e). An individual patient meta-analysis of the four cohorts (*n* = 649) was performed and there was low heterogeneity between the cohorts (*χ*^2^ = 3.5, *p* = 0.32). Patients with higher FKBPL levels had a significantly longer PFS from diagnosis (HR = 1.22, 95% CI 1.03–1.44 and *p* = 0.02) in the unstratified analysis (Fig. 6f), but significance was not reached in the stratified analysis (*p* = 0.07). The median FKBPL histoscore value over the four cohorts was 165 (interquartile range 146–186); however, cohort III had a significantly higher median at 190 and had a large number of censored events and thus was considered an outlier. Therefore, a second meta-analysis of cohorts I, II and IV was conducted (*n* = 550). There was a significant association between higher FKBPL levels and PFS from diagnosis in both the stratified (HR = 1.23, 95% CI 1.02, 1.47 and *p* = 0.03) and unstratified analysis (HR = 1.27, 95% CI 1.06, 1.52 and *p* = 0.009; Fig. 6g).

## Discussion

The majority of ovarian cancer patients relapse after standard treatment and this has been partially attributed to the CSC subpopulation. HGSOC therefore remains a disease of unmet clinical need, and here, for the first time, we evaluate a FKBPL peptide fragment, ALM201, to dual-target HGSOC stem cells and tumour angiogenesis.

One of the challenges with studying new therapeutics for HGSOC is determining the histopathological origin of the most commonly used cell lines. OVCAR3 and Kuramochi cells contain the major oncogenes and tumour-suppressor genes associated with HGSOC and are most likely to resemble the disease and were therefore used for the majority of the in vitro and in vivo experiments.^32^ ALM201 clearly demonstrated in vitro anti-CSC efficacy, by using both tumoursphere assays and flow cytometry in the OVCAR3 cell line monolayer (Figs. 1c, 2a–d). The Kuramochi cell line did not form tumourspheres or contain a CD44^+^/CD117^+^ cell population and also had a reduced ALDH^+^ subpopulation, thus indicating a lower CSC subpopulation in vitro. However, following treatment with ALM201, there was a decrease in spheres produced from PGCCs in vitro and a nonsignificant reduction in the ALDH^+^ population in the Kuramochi cell line (Figs. 1g, 2e).

FKBPL and its peptide derivatives have previously shown potent anti-angiogenic activity resulting in a tumour growth delay in a range of xenografts studies, potentially through the cell surface receptor CD44.^20,22,24^ However, for the first time, we observed no tumour growth delay in the OVCAR3 xenografts after treatment with ALM201 (Fig. 3b). Ex vivo analysis of the xenografts by IHC revealed limited blood vessels and extensive VM (Fig. 3g–i). In vitro tubule formation assays suggested that ALM201 had no effect on inhibiting VM channels in the OVCAR3 cells (Fig. 3j). In summary, the paucity of blood vessels, and the high level of VM within the OVCAR3 xenografts, is a likely explanation for ALM201’s lack of anti-angiogenic efficacy in this xenograft model.

Angiogenesis is regarded as an essential hallmark of cancer; however, non-angiogenic tumours have been reported to occur in brain,^42^ liver metastasis^43,44^ and lymph node metastasis.^45,46^ Gene expression analysis in angiogenic and non-angiogenic non-small cell lung cancer (NSCLC) samples suggests that in non-angiogenic tumours, hypoxia leads to an increased activation of the mitochondrial respiration chain and rapid tumour growth.^47^ Indeed, the OVCAR3 xenografts had a more rapid tumour growth, compared with the angiogenic Kuramochi xenografts (Figs. 3a, b, 4a, b). Moreover, there is emerging evidence in the literature that the non-angiogenic growth of tumours is responsible for both the intrinsic or acquired resistance to anti-angiogenic treatment.^48,49^ Here, for the first time, we describe an ovarian cancer xenograft that is dependent upon VM as opposed to classical angiogenesis. On the other hand, the Kuramochi cell line formed well-vascularised xenografts in vivo, and treatment with ALM201 resulted in a significant tumour growth delay (Fig. 4b). Analysis of the xenografts showed an extensive blood vessel network consistent with high expression of angiogenesis-related genes in Kuramochi cells (Fig. 5a). Kuramochi xenografts treated with ALM201 had decreased CD31^+^ blood vessels (Fig. 4d), in line with our previous studies with recombinant FKBPL and AD-01.^21,24^ Furthermore, the in vivo gold standard limiting dilution assay clearly demonstrated that ALM201 significantly decreased the tumour-initiating potential by ten-fold in Kuramochi xenografts (Fig. 4f). This result has significant clinical relevance since therapies against CSCs are a very active area of research, and there are comparatively very few agents that specifically target HGSOC stem cells. Overall, ALM201 had a potent anti-CSC efficacy in the Kuramochi cells in vivo and no effect on the OVCAR3 CSC population. This suggests that microenvironmental components are drastically different between the two different tumour xenografts, not surprising given the dramatic differences in tumour vascularisation in these tumour types.

These results further highlight the clinical need to stratify patients even within the same subtype of ovarian cancer. Four molecular subtypes within the umbrella of HGSOC (C1/mesenchymal, C2/immune, C4/differentiated and C5/proliferative) have been identified by gene expression profiling.^50^ Survival is statistically different between the subtypes: best in the immunoreactive type and worst in the proliferative or mesenchymal subtypes.^51^ The OVCAR3 and the Kuramochi cell lines are both indicative of the HGSOC subtype although the main drivers of in vivo tumour growth are clearly very different. In the clinic, treatment with ALM201 or any other anti-angiogenic therapy, in patients with HGSOC tumours with similar properties to the OVCAR3 subtype is likely to be ineffective. On the other hand, highly vascularised tumours, similar to the Kuramochi xenografts, are more likely to respond well to anti-angiogenic therapies, and encouragingly, ALM201 also exhibited a potent anti-CSC effect. Bais et al. recently demonstrated that higher microvessel density was predictive for response to bevacizumab in a Phase 3 clinical trial (GOG-0218).^52^ This may prove to be a simple and effective way to stratify patients likely to respond to anti-angiogenic therapy in HGSOC.

Intriguingly, ALM201 inhibited OVCAR3 CSCs in vitro; however, there was no effect on the CSC subpopulation in the OVCAR3 xenograft (Figs. 1c, 2a–d, 3c, d). Analysis of the Kuramochi and OVCAR3 xenografts showed that there were substantial differences in IL-6 and IL-8 at both mRNA and protein level. Analysis of the xenografts by using mouse and human ELISA revealed that only human IL-6 and IL-8 could be detected, thus suggesting that their source is tumour derived, rather than being from the endogenous mouse microenvironment. IL-6 and IL-8 levels were significantly elevated in vivo in the OVCAR3 xenografts compared with the monolayer, and no difference was observed between Kuramochi cells grown as monolayers or xenografts (Fig. 5c, d). We hypothesise that enrichment of the cytokines in OVCAR3 xenograft is a possible contributing factor to the inability of ALM201 to decrease stemness in vivo, whilst being effective in vitro where levels were substantially lower. Indeed, addition of recombinant IL-6 to in vitro OVCAR3 tumoursphere assays abrogated the ability of ALM201 to decrease TFE (Fig. 5e). The principal signalling pathway of IL-6 is STAT3, and for the first time, we demonstrated that ALM201 reduces activation of STAT3 in OVCAR3 cells (Fig. 5g, h). Future studies using fresh clinical samples are required to further investigate the role of IL-6- mediated resistance to FKBPL-based therapies.

We have provided evidence that FKBPL’s clinical peptide, ALM201, is a novel anti-CSC agent and a potent angiogenic inhibitor in vascularised HGSOC via STAT3 signalling. The current study will greatly enhance the clinical utility of this agent during its subsequent clinical development. In particular, we would suggest that well-vascularised tumours, with low IL-6, might be most responsive to its dual anti-angiogenic and anti-CSC activity, although this will need to be further validated in fresh clinical samples. Furthermore, we have demonstrated that high FKBPL levels were associated with an increase in PFS. These data indicate that FKBPL has potential as a novel prognostic biomarker in HGSOC, a cancer with no universally accepted biological prognostic biomarkers. Finally, we have provided further evidence that a number of different subtypes exist under the remit of HGSOC, with Kuramochi xenografts displaying extensive vascularisation and the OVCAR3 xenografts representative of ‘immune’ subtypes.

## Supplementary information

Supplementary Material

## Data Availability

All data generated or analysed during this study are included in this published article [and its additional files]. The authors can confirm that additional data are available on reasonable request.
