# Supplementary material for: FKBPL-based peptide, ALM201, targets angiogenesis and cancer stem cells in ovarian cancer
Source: Br J Cancer. 2019 Nov 27;122(3):361–71. doi: 10.1038/s41416-019-0649-5 (PMC7000737; doi:10.1038/s41416-019-0649-5)
Supplement: Supplementary file 1 — Supplementary Material [file 41416_2019_649_MOESM1_ESM.docx]

**Supplementary Materials and Methods**

**Cell culture**

OVCAR3 and Kuramochi cells were obtained from the American Type Culture Collection and were authenticated by short tandem repeat (STR) profiling carried out by the suppliers. Routine testing ensured cells were *Mycoplasma*-free. A2780, PE-01 and PE-04 cells were a kind gift from Dr Fiona Furlong, QUB. All cell lines were maintained in Roswell Park Memorial Institute (RPMI) 1640 medium (Invitrogen, UK) supplemented with 10% foetal bovine serum (FBS) (Invitrogen, UK) and used up to passage 20. All experiments were carried out at 37°C in a humidified atmosphere of 95% O_2_/5% CO_2_.

**Flow-cytometric analysis**

Following 72 h incubation with ALM201, OVCAR3 and Kuramochi cells were resuspended at 1 × 10^6^ in 100 μL sorting buffer (PBS containing 0.5% bovine serum albumin, 2 mmol/L EDTA) and incubated with pre-conjugated primary antibodies, CD44-APC (1:10; BD Pharmingen), and CD117-PE (1:20; BD Pharmingen) for 45 min at 4°C. The cells were washed in PBS and centrifuged at 800 × g for 2 min. For analysis, cells were resuspended in 500 μL of sorting buffer and fluorescence was measured using FACSCalibur and analyzed using WinMDI 2.8 or FlowJo 5.

**ALDEFLUOR assay**

Following 72 h incubation with ALM201, OVCAR3 and Kuramochi cells were analysed as described previously in (23).

**Primary tumor cell isolation**

Solid ovarian and omental samples were removed during cytoreduction surgery and were collected from ovarian cancer patients with fully informed consent (NIB13-0073; Northern Ireland Biobank, Supplementary Table 3) and minced to < 1mm^3^ using a scalpel. The tissue pieces were transferred to a gentleMACS C tube (Miltenyi Biotec, UK) containing RPMI with 300 U/ml collagenase and 100 U/ml hyaluronidase (Stem Cell Technologies, Canada) and 1% penicillin/streptomycin (Sigma-Aldrich, UK). The tissues were homogenised on the gentleMACS Dissociator (Miltenyi Biotec, UK) and incubated in an orbital incubator at 37°C for 2 h. After incubation, tissues were further homogenised and strained through a 200 µM filter (Miltenyi Biotec, UK) using a 5 ml syringe plunger to massage though tissue. This process was repeated with 100 µM, 70 µM and 40 µM filters (Miltenyi Biotac, UK). The cells were resuspended in Red Blood Cell Lysis buffer (Roche, UK) for 5 minutes at 37°C. Cells were resuspended in ice cold PBS and a cell count performed using a hemocytometer and trypan blue and cells were then used in a tumorsphere assay. Clinical information for each anonymized sample was received from the Northern Ireland Biobank including age, specimen histology, tumor grade and treatment information (Supplementary Table 3). The study was conducted in accordance with the Declaration of Helsinki.

**Induction of Polyploid Giant Cancer Cells (PGCCs)**

Cells were cultured in complete medium until 80 – 90% confluency. Cobalt chloride (CoCl2) solution (Sigma – Aldrich, UK) was added to the media to give a final concentration of 450 μM for 72 h. Following CoCl_2_ treatment, almost all regular sized cells were dead and PGCCs survived.

**PGCCs spheroid assay**

Kuramochi derived PGCCs were trypinised and counted using a hemocytometer. 1x10^3^ cells were mixed with phenol red free Matrigel (BD Bioscience, UK) and tumorsphere media at a 1:1 ratio in a total volume of 200 μl and seeded in a 24 well plate. The cell-Matrigel mixture was allowed to solidify at 37°C for 10 mins before 0.5 ml of tumorsphere media was added. PBS or ALM201 (100 nmol/L) was added directly to the well and the cells were incubated for 3 weeks at 37°C and 5% CO2/95%O_2_. The number of tumorspheres formed were counted by microscopy using a Nikon Eclipse TE300 (Japan) under 4X magnification. A spheroid was defined as a sphere colony > 50 µm.

**Clonogenic assay**

Methods were described previously (1). ALM201 treatment was added on day 2. Morphologically distinct colonies, representing holoclones, meroclones, and paraclones were counted manually (2).

**Tubule Formation Assay**

Growth factor reduced Matrigel (BD Bioscience) was added to the well of a µ-Slide Angiogenesis (Ibidi, Germany) and polymerised at 37°C for 30 min. 2 x 10^4^ OVCAR3 or Kuramochi cells were applied to the upper well in serum free RPMI and PBS or ALM201 (100 nmol/L) added. Slides were incubated at 37°C for 20 h. Five random images (x 10) were taken and the number of tubule loops per image counted manually.

**Polymerase Chain Reaction**

RNA was extracted following the standard protocol for Trizol reagent (ThermoFisher, UK). Xenografts were homologized in Trizol reagent with metallic beads (TissueLyserTL, Qiagen). The quality and quantity of the RNA was analysed using the NanoDrop Spectrophotometer and complementary DNA was transcribed with M-MLV Reverse Transcriptase (Invitrogen, UK). Validated Taqman mono hydrolysis PCR probes (Roche, UK) were used as previously described (3). Taqman Probes Master Mix (Roche, UK) was used for cDNA amplification using a Lightcycler 480 Instrument.

**ELISA**

Protein from xenografts was isolated using RIPA buffer, quantified using the Bradford assay and diluted to 3 µg/ml. Human and murine IL-6 and IL-8 (or KC) were quantified using DuoSet ELISA kits according to manufacturer’s instructions (R&D Systems, USA).

**Western Blot**

OVCAR3 cells were treated with ALM201 (100 nM) and/or IL-6 (50 ng/ml; Peptrotech) as per experimental conditions. Protein was extracted with RIPA buffer and protein quantified using BSA assay. Lysates were run on NuPage Gels and transferred using semi dry blotter. Membranes were probed with phospho STAT3(Tyr705) (Cell Signalling), STAT3 (Cell Signalling) and αβ tubulin (Cell signalling). Whole cell lysates from OVCAR3, OVCAR4, Kuramochi, PE01, PE04 cells were obtained membranes probed using FKBPL (Proteintech), RBCK1 (Abcam) and USP-19 (Abcam).

**Immunohistochemistry**

Xenografts were halved and one half fixed in 10% formaldehyde (Sigma-Aldrich, UK) for 24 h, paraffin embedded and 3 µm sections were mounted onto poly-L-lysine slides (Sigma-Aldrich, UK). The sections were deparaffinised, blocked in 3% hydrogen peroxide and antigen retrieval was performed using a pressure cooker. Slides were incubated with CD31 (1:150 dilution, Abcam, UK; ab28364) antibody overnight at 4°C, washed in TBT plus Tween 20 and incubated for 30 min at room temperature with Dako Envision^+^ system HRP Labelled Polymer Anti Rabbit antibody (Dako, Denmark). Vessels were visualised with DAB^+^ chromogen substrate (Dako, Denmark). For PAS staining, slides were immersed in periodic acid solution (PAS) (Sigma-Aldrich, UK) for 5 min at room temperature, rinsed in distilled water and immersed in Schiff’s reagent (Sigma-Aldrich, UK) for 15 min at room temperature. Slides were counterstained with haematoxylin and dehydrated, cleared and mounted using Ventana Discovery XT Immunostainier (Ventana Medical Systems In, USA). To assess microvessel density (MVD), forty representative images were randomly selected from scanned images of tissue sections (Path XL). A blinded assessor quantified the number of vessels per field of view.

**RNA sequencing**

100 ng of total RNA was used to generate RNA libraries using the stranded mRNA library preparation kit on the Neoprep library generation (Illumina Ltd.) platform as per manufacturer’s instructions. The generated libraries were quantified and normalized as part of the library generation protocol on the Neoprep system. The quality of the library prior to sequencing was assessed using a High-sensitivity Bioanalyzer assay (Agilent Technologies). Subsequently, the libraries were sequenced in a 2 x 75bp fashion on the NextSeq 500 platform to generate fastq files for further bioinformatic analysis.

**Database**

Expression array data was evaluated using the online tool termed the Kaplan–Meier Plotter (<http://www.kmplot.com/ovar>) as described (4). The probe set used for *fkbpl* analyses was 219187_at. Query parameters were: overall survival, split patients by median, auto-select best cut-off, and all follow up threshold. Signal range of the probe was 25 – 1232 and the auto-cutoff value was 291. Restriction analyses were stage (all), histology (serous), grade (all), debulk (all), and chemotherapy treatments (all). 1207 patient samples were analyzed.

**Statistical analysis**

Data presented are a mean of at least 3 independent experiments ± standard error (SE). One-way analysis of variance (ANOVA) or two-tailed t tests were used to assess differences between various treatments. Kaplan-Meier curves were constructed for overall survival and progression free survival (PFS). Overall survival calculated from the date of diagnosis until the date of death from any cause. PFS was calculated from the date of diagnosis until the date of first progression or death from any cause. A Cox proportional hazards model was used to obtain hazard ratios, 95% confidence intervals and associated p-value. To assess the effect of FKBPL across cohorts a hazard ratio plot was constructed and the Cochran’s Q statistic was used to assess the level of heterogeneity between cohorts. A receiver operator curve (ROC) analysis for predicting progression/death events was used to determine a suitable cutpoint for FKBPL. Statistical significance was determined by the P values less or equal to 0.05; *, P < 0.05; **, P < 0.01; ***, P < 0.001.

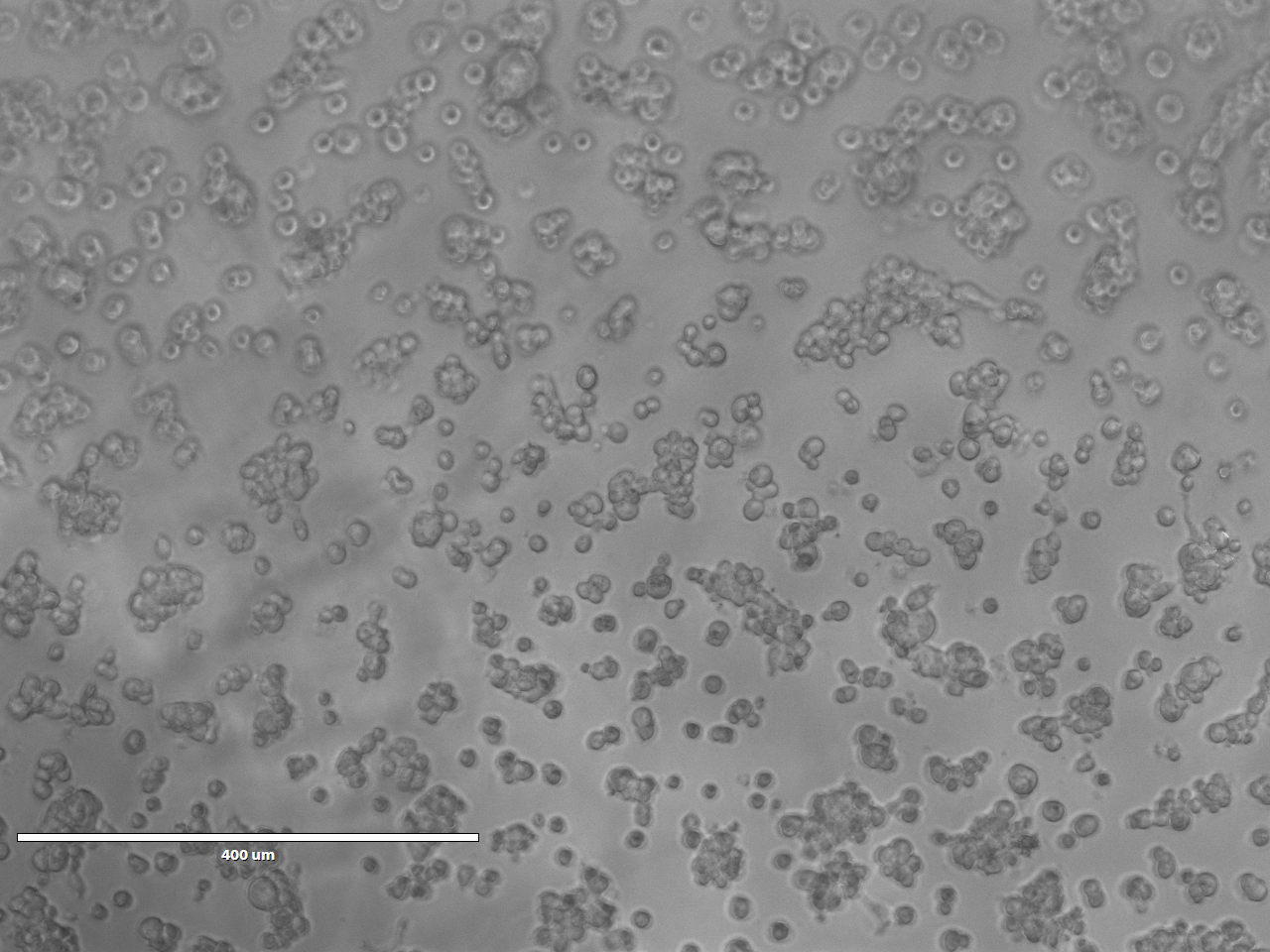


**Supplementary Figure 4**

**Supplementary Figure Legends**

Supplementary Figure 1 FKBPL, USP19 and RBCK1 protein expression across a panel of ovarian cancer cell line Average protein expression of FKBPL, USP19, RBCK1 in OVCAR3, OVCAR4, Kuramochi, PE01, PE04 and A2780 analsysed by western blot. Data points are mean ± SEM. *n* = 3. *, *P* < 0.05; **, *P* < 0.01; ***, *P* < 0.001 (one way ANOVA).

Supplementary Figure 2 ALM201 treatment significantly reduces tumoursphere formation in samples from HGSOC patients with no previous chemotherapy treatment.

(A) Fresh ovarian HGS patient samples obtaining after cytoreduction surgery that did not receive chemotherapy (Sample ID; 02316 0020, 0242F 0020, 02E180020). (B) Fresh ovarian HGS patient samples obtaining after cytoreduction surgery that received neo-adjuvant chemotherapy (Sample ID; 026C 0020, 03878, 032390019). The number of tumourspheres (> 50 μm) per well were counted manually and divided by number of cell seeded to calculate the tumoursphere forming efficacy (TFE). Data points represent mean the ± SEM; n=3 (one way ANOVA).

Supplementary Figure 3 ALM201 activity in decreasing tumorsphere formation in non-high grade serous ovarian cancer cell line and fresh patient samples

(A) ALM201 significantly reduced tumorsphere formation in A2780 ovarian cell line (n=3). (B) ALM201 treatment reduces tumorsphere formation in clinically derived metastatic endometrioid adenocarcinomia primary omentum tissue. The ovarian tissue did not form tumorspheres. (C) ALM201 treatment may promote tumorsphere formation in clinically derived metastatic mucinous primary ovarian and omentum tissue. (D) ALM201 treatment reduces tumorsphere formation in clinically derived metastatic clear cell primary omentum tissue. (E) ALM201 treatment reduces tumorsphere formation in serous borderline sample.

Supplementary Figure 4 Kuramochi cells do not form tubules *in vitro*

A single cell suspension was seeded onto growth factor reduced media in serum free media and observed for tubule formation. A representative image of Kurmaochi cells incubated for up to five days without tubule formation.

Supplementary Figure 5 A log- fraction plot of Kuramochi limiting dilution assay.

A log-fraction plot of the limiting dilution model fitted to the data in Fig. 4F. The slope of the line is the log-active cell fraction. The dotted lines give the 95% confidence interval.

Supplementary Figure 6 RNA sequencing analysis of Kuramochi and OVCAR3 cell line monolayers

Heat map of genes upregulated (red) in Kuramochi cell line compared to the OVCAR3 cell line by RNAseq analysis in the (A) p38/MAPK pathway (B) TGF beta pathway (C) mTOR pathway (D) NOD like receptor signaling.

Supplementary Figure 7 Knockdown of *Fkbpl* in OVCAR3 cells results in an upregulation of mRNA *Nfκb1* and *Nanog*

(A) Transient siFKPBL knockdown in OVCAR3 cells results in an upregulation of mRNA (B) *Nfκb1* and (C) *Nanog*. Data points are mean ± SEM. *n* ≥ 3. *, *P* < 0.05; **, *P* < 0.01; ***, *P* < 0.001 (unpaired student t test)

Supplementary Figure 8 Receiver operated curve (ROC) analysis of ovarian cohort I and II

The cut off point on the ROC curve that minimises the distant to the (0,1) point is an FKBPL score of 190.

**Supplementary Tables**

Table 1 ARRIVE guidelines


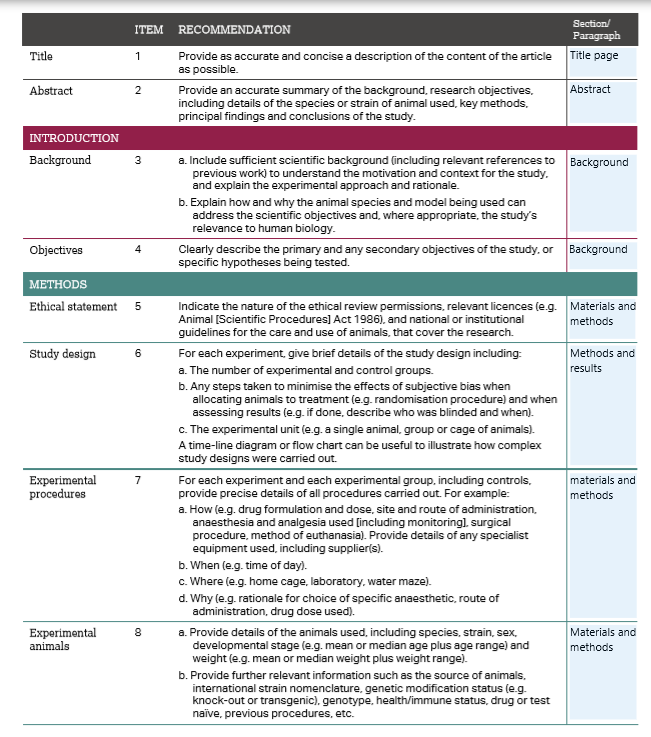


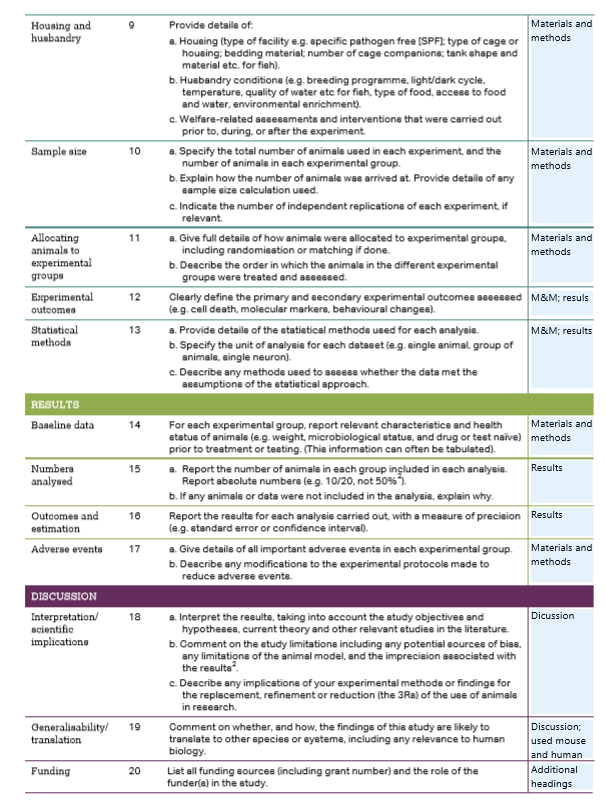


Table 2 Patient characteristics of ovarian cancer cohorts in TMA analysis

| **Patient characteristic** | **Cohort** | | | |
| --- | --- | --- | --- | --- |
|  | **I: 08-001 TB** | **II: 08-004 Ext** | **III: TCD** | **IV: WMH** |
|  | **N (%)** | **N (%)** | **N (%)** | **N (%)** |
| **Total with FKBPL scores and analysed** | 177 | 193 | 99 | 180 |
| **Age** |  |  |  |  |
| **Median (Interquartile range)** | 61 (52-70) | 63 (54-70) | 60 (48-72) | 61 (52-68) |
| **Range** | 34-90 | 23-90 | 23-82 | 22-91 |
| **Stage** |  |  |  |  |
| **1/2** | 25 (14) | 10 (5) | 0 | 16 (9) |
| **3** | 130 (74) | 163 (85) | 80 (81) | 145 (81) |
| **4** | 22 (12) | 20 (10) | 19 (19) | 19 (10) |
| **Neoadjuvant chemotherapy** |  |  |  |  |
| **No** | 136 (77) | 165 (85) | 0(99) | 179 (99) |
| **Yes** | 41 (23) | 28 (15) | 0(99) | 1 (1) |
| **Optimal surgical debulking** |  |  |  |  |
| **No residual disease** | 37 (21) | 0 | 88 (89) | 38 (21) |
| **Visible residual disease** | 140 (79) | 193 (100) | 8 (8) | 142 (79) |
| **Unknown** | 0 | 0 | 3 (3) | 0 |
| **FKBPL histoscore** |  |  |  |  |
| **Median (interquartile range)** | 150 (100-200) | 145 (100-200) | 190 (125-239) | 175 (103-213) |
| **Range** | 0-300 | 0-300 | 0-300 | 0-300 |
| **<190** | 110 (62) | 133 (69) | 46 (46) | 104 (58) |
| **>= 190** | 67 (38) | 60 (31) | 53 (54) | 76 (42) |

Table 3 Clinical data of primary ovarian tumors obtained after cytoreduction surgery

| **Patient ID** | **Specimen** | **Pathology subtype** | **Stage** | **Age** | **Previous treatment for ovarian cancer** |
| --- | --- | --- | --- | --- | --- |
| 023160020 | Omentum (ovarian capsule intact) | High grade serous ovarian cancer | IIA | 49 | N/A |
| 0242F0020 | Omentum | High grade serous ovarian cancer | IIIC | 60 | N/A |
| 02E180020 | Omentum | Fallopian Tube High Grade Serous | Unknown | 75 | N/A |
| 026C0020 | Ovary | Fallopian Tube High Grade Serous | IIIC | 67 | Four cycles of chemotherapy prior to surgery |
| 03878 | Omentum | Fallopian Tube High Grade Serous | IIIC | 66 | Incomplete cyroreduction four months prior. Four cycles of chemotherapy with partial response prior to second cyto reduction |
| 032390019 | Omentum | High grade serous ovarian cancer | IIIC | 61 | Three cycles of paclitaxel/carboplatin four months prior to surgery |
| 035C00019 | Omentum | Endometrioid Adenocarcinomia metastatic carcinoma | IIIB | 32 | N/A |
| 0218F0020 | Omentum | Metastatic ovarian clear cell | Unknown (resectable disease) | 77 | N/A |
| 02F550020 | Ovary | Clear Cell carcinoma | IC2 | 52 | N/A |
| 02422 0019 | Ovary | Serous Borderline  Non-invasive implants | IIIA2 | 59 | N/A |
